# Cigarette, cigar and pipe smoking, passive smoke exposure, and risk of pancreatic cancer: a population-based study in the San Francisco Bay Area

**DOI:** 10.1186/1471-2407-11-138

**Published:** 2011-04-15

**Authors:** Gregory J Tranah, Elizabeth A Holly, Furong Wang, Paige M Bracci

**Affiliations:** 1 California Pacific Medical Center Research Institute, CA, 94107, USA; 2 Department of Epidemiology and Biostatistics, University of California San Francisco, CA, 94143, USA

## Abstract

**Background:**

To examine the influence of cigarette, cigar and pipe smoking, cessation of cigarette smoking and passive smoke exposure on the risk of pancreatic cancer.

**Methods:**

Exposure data were collected during in-person interviews in a population-based case-control study of pancreatic cancer (N = 532 cases, N = 1701 controls) in the San Francisco Bay Area. Odds ratios (ORs) were adjusted for potential confounders.

**Results:**

The adjusted odds ratio (OR) of pancreatic cancer among current smokers was 1.9 (95% confidence interval (CI), 1.4-2.7). A significant, positive trend in risk with increasing pack-years of smoking was observed (P-trend <0.0001). Compared with participants who continued to smoke, former smokers had no statistically significant elevation in risk of pancreatic cancer 10 years after smoking cessation, with risk reduced to that of never smokers regardless of prior smoking intensity. Both men and women experienced similar increased risk of pancreatic cancer with increasing smoking duration. Cigar and pipe smoking and exposure to passive smoke were not associated with pancreatic cancer.

**Conclusions:**

Cigarette smoking is associated with an increased risk of pancreatic cancer. Smokers who had quit for ≥10 years no longer experienced an increased risk. Future work will help to determine the effect of declining smoking rates on pancreatic cancer incidence.

## Background

Pancreatic cancer is the fourth leading cause of death from cancer among men and women in the United States and is expected to result in 43,140 new cases and 36,800 deaths in 2010 [1]. Due to its aggressiveness and a lack of early detection methods, pancreatic cancer is often metastatic in patients at the time of diagnosis resulting in a 5-year relative survival rate for all stages of <5%. The incidence of pancreatic cancer varies by age, sex, and race - and survival differences by race have been observed [2-6].

Of the several modest risk factors that have been identified for pancreatic cancer, including obesity, diabetes, physical inactivity, chronic pancreatitis and dietary factors [7-12], cigarette smoking has been reported most consistently [2,13-17]. In a large meta-analysis [14] current cigarette smoking was associated with an overall relative risk (RR) of pancreatic cancer of 1.7 whereas current cigar and pipe smoking was associated with an approximate RR of 1.5. There was evidence of a dose-response relationship between smoking and pancreatic cancer [14] although the risk for those who had quit for10 or more years was not different from that of nonsmokers [14]. A recent pooled analysis from the Pancreatic Cancer Cohort Consortium [17] identified an odds ratio (OR) of 1.8 for current cigarette smoking and that risk was similar to that for never smokers after more than 15 years of smoking cessation.

It has been estimated that 25-30% of pancreatic cancer cases in the United States are related to cigarette smoking [18,19]. Because of their public health implications, the effects of smoking cessation on pancreas cancer risk are important to understand. In the present population-based, case-control study of pancreatic cancer we examined cigarette, cigar and pipe smoking as risk factors for pancreas cancer. We quantified the relationship between cigarette smoking and pancreatic cancer and estimated the effects of smoking duration, pack-years smoked, cigarettes smoked/day and cigarette smoking cessation on risk of developing pancreatic cancer. Lastly, we assessed the effects of passive cigarette smoke exposure on the risk of pancreatic cancer.

## Methods

### Study Participants

Detailed methods for this case-control study have been published [20-24]. Briefly, a population-based case-control study of pancreatic cancer was conducted in six San Francisco Bay Area counties (Alameda, Contra Costa, Marin, San Francisco, San Mateo, and Santa Clara). Cases with primary adenocarcinoma of the exocrine pancreas were identified using rapid case ascertainment by the Northern California Cancer Center (Union City, CA) with a goal to identify patients in the study area within one month of diagnosis. Eligible cases were newly diagnosed between 1995 and 1999 with adenocarcinoma of the exocrine pancreas who were between 21 and 85 years old, resided in one of the six Bay Area counties listed above, were alive at the time of the first attempted contact, and could complete an interview in English. There were 65 out-of-area pancreatic cancer patients identified through clinical records at the UCSF Medical Center who were eligible to participate because they met all study criteria other than their place of residence at the time of diagnosis. Diagnoses for out-of-area patient participants were confirmed using patients' pathology reports and medical records. A total of 532 eligible cases completed the interview for a 67% response rate. The refusal rate was 8% among cases. No eligible patients with completed interviews were excluded from this analysis. Patient diagnoses were confirmed by participants' physicians and by the Surveillance, Epidemiology, and End Results abstracts that included histologic confirmation of disease.

Control participants for the in-area cases were identified within the six San Francisco Bay Area counties using random-digit dial (RDD), and were frequency-matched to cases by sex and 5-year age group in an approximate 3:1 ratio. Eligibility criteria were identical for case and control participants except for pancreatic cancer status. Control identification for those >65 years of age was supplemented by random selection from Health Care Finance Administration lists (now the Centers for Medicare and Medicaid Services) for the six Bay Area counties. Out-of-area controls also were identified by RDD, were frequency matched to out-of-area cases by residential telephone area code and prefix, and by sex and 5-year age group. A total of 1,701 eligible control participants completed the study interview for a 67% response rate [21]. The refusal rate among controls was 9%.

### Interviews

Detailed in-person interviews were conducted in the homes of the participants or at a location of their choice for Bay Area participants and by telephone interview for out-of-area participants. No proxy interviews were conducted. The University of California San Francisco Committee on Human Research approved the study protocols and procedures. Written informed consent was obtained from each study participant before interview. Primary topics included on the questionnaire were: demographic factors, detailed job and occupational history, use of tobacco products and alcohol consumption, medical history including allergies, diabetes, pancreatitis and gallbladder disease, family medical history, anthropometric data and a detailed assessment of diet including average consumption of specific foods, vitamin supplement use and dietary modifications.

Race/ethnicity was based on self-report and was broadly defined as Caucasian, black/African-American, Asian, and Hispanic (black or white). Nine cases and 33 controls who were of mixed race/ethnicity were classified as "other race/ethnicity" for these analyses. Participants were defined as smokers if they had smoked >100 cigarettes in their lifetime. Former smokers were defined as those who had ceased smoking one year or more prior to diagnosis or interview. Those who had quit less than one year prior to diagnosis/interview were considered current smokers. Participants were considered cigar and/or pipe smokers if they had ever smoked pipes and/or cigars for >6 months. Body mass index (BMI; weight in kilograms divided by height in meters squared) was based on self-reported height and usual adult weight and was categorized based on quartiles of the distribution among controls by sex as follows: men: <23.1; 23.1-<25.1; 25.1-<27.1; >27.1 kg/m^2^; and women: <21.5; 21.5-<23.4; 23.4-<25.8; >25.8 kg/m^2^. Participants were defined as consumers of alcohol if they had ever had at least one alcoholic drink per month.

### Statistical analysis

Unconditional logistic regression was used to obtain odds ratios (ORs) as estimates of relative risks (hereafter called risk) and 95% confidence intervals (CIs). All models were adjusted for age in 5-year groups, sex, education level (≤12 years, 12-16 years, >16 years), race/ethnicity (Hispanic white, non-Hispanic white, black or African-American, Asian, other), diabetes diagnosis, pancreatitis diagnosis, gallbladder disease (gallstones or gallbladder inflammation), alcohol intake and BMI [24-28]. Models for cigar/pipe smoking and passive smoke exposure were further adjusted for cigarette smoking status (never, past and current). In the combined analysis of smoking intensity and years of smoking cessation, similarity of ORs was tested using the Breslow-Day test for homogeneity.

## Results

A total of 532 cases and 1,701 control participants completed the interview. There were 16 cases and 73 controls who reported smoking cigars or pipes but not cigarettes leaving a total of 516 cases and 1628 controls who were included in the final analyses of cigarette smoking. Distribution by age, education level, race, ethnicity, sex, BMI, diabetes diagnosis, pancreatitis diagnosis, gallbladder problems and alcohol intake for total pancreas cancer cases and controls are presented in Table 1. The mean age for pancreas cancer cases and control participants was 65 and 64 years, respectively. In general, cases were somewhat less educated and a greater proportion were men, black, had a high BMI (men only) and were more likely to have been diagnosed with diabetes, pancreatitis or gallbladder disease.

**Table 1:** Characteristics of pancreatic cancer (PanCA) cases and controls in a population based study in the San Francisco Bay Area

		PanCA	Controls
Characteristic		N = 532	N = 1701
Age (years)	Mean (s.d.)	65 (11)	64 (11)
			
Education (years)		n (%)	n (%)
	1-12	235 (44)	534 (31)
	12-16	200 (38)	754 (44)
	Over 16	97 (18)	413 (24)
Race/ethnicity			
	White, non-Hispanic	417 (78)	1357 (80)
	White, Hispanic	25 (5)	114 (7)
	Black	46 (9)	78 (5)
	Asian	35 (7)	119 (7)
	Other	9 (2)	33 (2)
Sex			
	Men	291 (55)	883 (52)
	Women	241 (45)	818 (48)
			
Usual adult BMI			
Men	<23.1	48 (17)	222 (25)
	23.1-<25.1	70 (24)	223 (25)
	25.1-<27.1	75 (26)	214 (24)
	27.1+	95 (33)	221 (25)
			
Women	<21.5	67 (28)	197 (24)
	21.5-<23.4	51 (21)	208 (26)
	23.4-<25.8	62 (26)	208 (26)
	25.8+	61 (25)	198 (24)
			
Diabetes diagnosis			
	Yes	69 (13)	152 (9)
	No	455 (87)	1538 (91)
			
Pancreatitis diagnosis			
	Yes	37 (7)	19 (1)
	No	491 (93)	1681 (99)
			
Gallstones or gallbladder inflammation			
	Yes	95 (18)	207 (12)
	No	432 (82)	1493 (88)
			
Alcohol (ever had at least one alcoholic drink per month)			
	Yes	447 (84)	1396 (82)
	No	85 (16)	305 (18)

### Smoking Status and Quantity

The OR for current smokers was 1.9 compared with never smokers (Table 2). Risk increased with increasing intensity of smoking that was assessed in three ways, as the number of cigarettes smoked per day, years smoked, and pack-years smoked (Table 2). Compared with nonsmokers, participants who smoked for at least 40 years had an OR of 1.8 and those who smoked at least 40 pack-years had an OR of 2.0. Analyses by sex and restricted race/ethnicity showed that men (p = 0.004) and women (p = 0.009) from all races/ethnicities, and that non-Hispanic whites (p = 0.0002) experienced increased risk with increasing duration smoked (data not shown). Smoking 40 or more years was associated with similar increases in risk for men (OR = 1.8, 95%CI = 1.2-2.9), women (OR = 1.8, 95%CI = 1.2-2.7) and non-Hispanic white men and women (OR = 1.8, 95%CI = 1.3-2.6) (data not shown). Sample sizes were too small to examine associations with cigarette smoking among other race and ethnicity groups. Smoking at least one cigar or pipe per month for over six months was not associated with pancreatic cancer (Table 2).

**Table 2:** Odds Ratios (OR) and 95% confidence intervals (CI) for pancreatic cancer (PanCA) associated with smoking status, intensity and duration, San Francisco Bay Area, California

		PanCA^a^	Controls^a^	
		N = 532	N = 1701	OR (95% CI)^b^
		n (%)	n (%)	
	Never smoked	163 (32)	650 (40)	1.0 (Ref.)
				
Cigarette Smoking				
	Former smoker^c^	241 (47)	781 (48)	1.1 (0.87 - 1.4)
	Current smoker^d^	112 (22)	197 (12)	1.9 (1.4 - 2.7)
			P-trend	0.0002
				
Ever smoked cigars and/or pipes^e^		100 (19)	334 (32)	0.88 (0.66 - 1.2)
				
Average cigarettes/day				
	<20	194 (38)	608 (37)	1.2 (0.92 - 1.5)
	20-<40	137 (27)	329 (20)	1.4 (1.0 - 1.9)
	40+	22 (4)	41 (3)	1.8 (0.99 - 3.1)
			P-trend	0.008
				
Years smoked				
	<20	177 (34)	637 (39)	0.9 (0.68 - 1.7)
	20-<40	64 (12)	144 (9)	1.3 (0.98 - 1.7)
	40+	112 (22)	197 (12)	1.8 (1.3 - 2.4)
			P-trend	0.0001
				
Pack years				
	<20	129 (25)	487 (30)	1.1 (0.81 - 1.4)
	20-<40	110 (21)	310 (19)	1.2 (0.90 - 1.6)
	40+	114 (22)	181 (11)	2.0 (1.5 - 2.8)
			P-trend	<.0001

^a ^The number of participants may vary because of missing data

^b ^Adjusted for: age, education, race, ethnicity, diabetes, pancreatitis, gallbladder disease, alcohol intake and body mass index

^c ^Includes smokers who quit ≥1 year prior to diagnosis/interview

^d ^Includes former smokers who quit within the immediate 1 year prior to interview

^e ^Includes cigar and pipe smokers who also smoked cigarettes and 16 cases and 73 controls who were cigar and/or pipe smokers who did not smoke cigarettes

### Smoking Cessation

The effects of smoking cessation were examined in mutually exclusive groups according to smoking duration (Table 3). Former smokers who quit ≥10 years prior to diagnosis or interview had an OR similar to that of nonsmokers (OR = 0.99, 95% CI = 0.77-1.3; data not in tables) whereas for former smokers who quit ≤10 years ago the OR was 1.6 (95% CI = 1.1-2.3). Relative to current smokers, risk diminished among former smokers who had quit 10-<15 years prior to diagnosis or interview. Former smokers who had quit for ≥15 years had no elevated risk of pancreatic cancer. We also conducted dichotomized analyses that were stratified by years since quitting to assess whether duration of cessation (<10 years, ≥10 years) modified the effect of lifetime or daily cigarette consumption on risk of pancreatic cancer (Table 4). Former smokers who quit ten or more years prior to diagnosis or interview had an OR similar to that of nonsmokers regardless of prior smoking intensity (Table 4). However, the effect of smoking cessation among those who quit fewer than ten years prior to diagnosis or interview varied somewhat by prior smoking intensity when compared to nonsmokers (Table 4). For example, the risk of pancreatic cancer for those who smoked for 40 or more years was similar to current smokers (OR = 1.8), but not for those who had smoked fewer than 40 years (OR = 1.3). However, confidence limits overlapped and tests for homogeneity showed that the estimated ORs were similar by smoking intensity or time of cessation (Table 4).

**Table 3:** Odds Ratios (OR) and 95% confidence intervals (CI) for pancreatic cancer (PanCA) associated with cigarette smoking cessation, San Francisco Bay Area, California.

		PanCA^a^	Controls^a^	
		N = 516	N = 1628	OR (95% CI)^b^
		n (%)	n (%)	
	Never smoked	163 (32)	650 (40)	1.0 (Ref.)
Years since last smoked^c^				
	>25	97 (19)	370 (23)	0.98 (0.72 - 1.3)
	20-<25	25 (5)	89 (5)	0.92 (0.55 - 1.5)
	15-<20	23 (4)	92 (6)	0.84 (0.5 - 1.4)
	10-<15	33 (6)	87 (5)	1.2 (0.75 - 1.9)
	<10	31 (6)	51 (3)	1.6 (1.1 - 2.3)
	Current smoker^d^	112 (22)	197 (12)	1.9 (1.4 - 2.7)

^a ^The number of participants may vary because of missing data

^b ^Adjusted for: age, education, race, ethnicity, diabetes, pancreatitis, gallbladder disease, alcohol intake and body mass index

^c ^Includes smokers who quit ≥1 year prior to diagnosis/interview

^d ^Includes former smokers who quit within the immediate 1 year prior to interview

**Table 4:** Odds Ratios (OR) and 95% confidence intervals (CI) for pancreatic cancer (PanCA) associated with cigarette smoking cessation in relation to frequency of use compared with nonsmokers, San Francisco Bay Area, California.

	Years since quitting^a^	
**Lifetime Cigarette Frequency of Use**^**c**^	<10	>=10
	**OR (95% CI)**^**b**^	**OR (95% CI)**^**b**^
<40 years smoked	1.3 (0.81 - 2.2)	1.0 (0.76 - 1.3)
>=40 years smoked	1.8 (1.1 - 2.9)	0.99 (0.56 - 1.7)
Test for Homogeneity^d ^(p-value)		0.29
		
<20 cigarettes/day	1.8 (1.2 - 2.8)	0.96 (0.71 - 1.3)
>= 20 cigarettes/day	1.3 (0.75 - 2.2)	1.0 (0.73 - 1.5)
Test for Homogeneity^d ^(p-value)		0.26
		
<40 packyears	1.8 (1.1 - 2.8)	0.96 (0.73 - 1.3)
>= 40 packyears	1.4 (0.83 - 2.3)	1.2 (0.74 - 1.8)
Test for Homogeneity^d ^(p-value)		0.45

^a ^The number of participants may vary because of missing data

^b ^Compared with nonsmokers. Adjusted for: age, education, race, ethnicity, diabetes, pancreatitis, gallbladder disease, alcohol intake and body mass index

^c ^Includes smokers who quit ≥1 year prior to diagnosis/interview

^d ^Breslow-Day test

### Passive Exposure to Smoking

Passive exposure to household and workplace smoke was not associated with an increased risk of pancreatic cancer (Table 5). Childhood household exposure (OR = 0.99), adult household exposure (OR = 1.2) and workplace (OR = 1.1) exposure were not associated with an increased risk of pancreatic cancer after adjustment for smoking behavior (never, past, current). The categories are not mutually exclusive, which allowed for the examination of exposure to multiple exposure environments (e.g. exposure at home and work). Analysis of mutually exclusive exposure environments (e.g. exposure at either home or work) did not yield significant associations with pancreatic cancer risk (data not shown).

**Table 5:** Odds Ratios (OR) and 95% confidence intervals (CI) for pancreatic cancer (PanCA) associated with passive exposure to cigarette smoke, San Francisco Bay Area, California.

		PanCA^a^	Controls^a^	
		n (%)	n (%)	OR (95% CI)^b^
Childhood household exposure^c^				
	Never	230 (43)	758 (45)	1.0 (Ref.)
	Ever	301 (57)	940 (55)	0.99 (0.80 - 1.2)
				
Adulthood household exposure^c^				
	Never	239 (45)	888 (52)	1.0 (Ref.)
	Ever	293 (55)	813 (48)	1.2 (0.96 - 1.5)
				
Adulthood workplace exposure^c^				
	Never	232 (45)	827 (48)	1.0 (Ref.)
	Ever	293 (55)	874 (45)	1.1 (0.86 - 1.3)

^a ^The number of participants may vary because of missing data

^b ^Adjusted for: age, education, race, smoking status (never, former, current), ethnicity, diabetes, pancreatitis, gallbladder disease, alcohol intake and BMI adjusted

^c ^Exposure environments are not mutually exclusive (i.e. participants could have been exposed in multiple environments)

## Discussion

The results from this population-based case-control study expand upon those of earlier case-control and cohort studies that have demonstrated the association between smoking and pancreatic cancer risk. The current estimation of the overall risk of pancreatic cancer among current smokers is consistent with the 1.6- to 1.9-fold increased risk reported by other investigators [14]. Our results support previous work that has identified a relationship between smoking intensity and duration and pancreatic cancer [14,17]. After adjustment for potential confounders and suspected risk factors, we observed a two-fold increased risk of pancreatic cancer among those who smoked 40 or more pack years and similarly elevated risks were observed for those who had smoked for more than 40 years or more than 40 cigarettes per day. Unlike previous studies reporting stronger smoking-related associations among women than among men [9,29,30], we observed similar risks for women and men. In contrast to a recent meta-analysis [14], we did not observe an increased risk of pancreatic cancer among current cigar and/or pipe smokers. However, limited sample size restricted our examinations to participants who ever smoked pipes and cigars and who also smoked cigarettes.

The effects of smoking cessation in our study population also were similar to previously reported results [14,17]. In the current study, the risk estimate among former smokers who quit 10 years prior to diagnosis or interview was similar to the decrease observed in the meta-analysis of 82 published studies [14]. To eliminate the combined effect of short and long-term duration of years since quit, we expanded upon these analyses by investigating duration of smoking cessation in mutually exclusive groups. Our results showed that former smokers who had quit fewer than 13 years prior to diagnosis or interview had a greater than 1.6 fold elevated risk of pancreatic cancer. In contrast, former smokers who had quit 10-<15 or ≥15 years prior to diagnosis or interview had lower magnitude ORs that were not different from unity. Participants who had stopped smoking for ten or more years prior to diagnosis or interview had no increased risk of pancreatic cancer relative to nonsmokers, regardless of prior smoking intensity. In contrast, the effect of smoking cessation among those who had quit fewer than ten years prior to diagnosis or interview varied by prior smoking intensity. These results are similar to those of a previous study that examined the combined effects of smoking cessation and prior smoking intensity [31]. Relative to current smokers, a consistent reduction in risk was observed for those former smokers who had quit for fewer than 10 to at least 25 years (in 5-year mutually exclusive groups).

Environmental tobacco smoke contains thousands of chemicals including dozens of known carcinogens [32] yet a link between passive or secondhand smoke exposure and pancreatic cancer has not been established [33,34]. In this study, passive exposure to household and workplace cigarette smoke among adults was not associated with an increased risk of pancreatic cancer, nor was childhood household exposure. We may not have expected to see an association with childhood or early adult exposure to passive smoke due to the long time interval to cancer development. This is supported by our results that demonstrate no increased risk for personal smoking after ten years of cessation.

Strengths of this study included the large sample size, the population-based design, similarity of response rates for the cases and controls, and the low case refusal rate of 8 percent. The primary reason for lost patients was aggressive disease and the high mortality rate of pancreatic cancer. Study design methods used to diminish potential selection bias included random-digit-dial to identify age-, sex- and county-matched controls from the same population from where the cases were obtained and rapid case ascertainment to identify all incident pancreatic cancer cases diagnosed in six Bay Area counties between 1994 and 1999. Trained experienced interviewers who were unaware of the study hypotheses conducted in-person interviews with participants to diminish interviewer bias and no proxy interviews were conducted to diminish recall and misclassification bias. Several limitations also should be considered when interpreting the results of this study. In particular, as in all case-control investigations, there may have been recall bias due to differential reporting of past smoking by cases versus controls because smoking could possibly have been known as a risk factor for pancreatic cancer among some individuals. However, cases and controls were equally likely to have quit smoking, suggesting that the likelihood that the observed associations were due to recall bias among the cases was low. In addition, the eligible cases who had died prior to interview may have been smokers disproportionately to nonsmokers and this may have affected the ORs resulting in bias toward the null. Multiple comparisons were made in this study, and some results could have been due to chance. However, this study was designed to test specific hypotheses related to smoking, and the outcomes of the analyses were generally consistent with previous studies supporting a causative role of cigarette smoking for pancreatic cancer.

## Conclusions

These data support evidence that cigarette smoking is a risk factor for pancreatic cancer. This investigation reinforces the results of earlier studies by demonstrating the benefits of smoking cessation on risk of pancreatic cancer and extends the earlier reports by: quantifying the relationship between cigarette smoking intensity and pancreatic cancer; estimating the modifying effects of smoking cessation by smoking duration, pack-years smoked, and cigarettes smoked per day on risk of developing pancreatic cancer; and assessing the effects of cigar and pipe smoking and passive cigarette smoke exposure on the risk of pancreatic cancer. These data along with the results from previous studies suggest that future work should focus on the effect that declining smoking rates will have on pancreatic cancer incidence.

## Competing interests

The authors declare that they have no competing interests.

## Authors' contributions

GJT conducted the data analysis; participated in data interpretation; and drafted the manuscript. EAH conceived of the study; participated in study design, coordination, and acquisition of data; participated in data interpretation; and revised the manuscript critically. FW participated in data analysis and interpretation. PMB participated in study design, coordination, and acquisition of data; participated in data analysis and interpretation; and revised the manuscript critically. All authors read and approved the final manuscript

## Pre-publication history

The pre-publication history for this paper can be accessed here:

http://www.biomedcentral.com/1471-2407/11/138/prepub
